# Sealing Efficacy of the Original and Third-Party Custom-Made Abutments—Microbiological In Vitro Pilot Study

**DOI:** 10.3390/ma15041597

**Published:** 2022-02-21

**Authors:** Igor Smojver, Roko Bjelica, Amir Ćatić, Ana Budimir, Marko Vuletić, Dragana Gabrić

**Affiliations:** 1 St. Catherine Specialty Hospital, 10000 Zagreb, Croatia; ismojver@gmail.com; 2 Department of Oral Surgery, School of Dental Medicine, University of Zagreb, 10000 Zagreb, Croatia; rbjelica@sfzg.hr (R.B.); mvuletic@sfzg.hr (M.V.); 3 Department of Fixed Prosthodontics, School of Dental Medicine, University of Zagreb, 10000 Zagreb, Croatia; catic@sfzg.hr; 4 Department of Dental Medicine, University Hospital Centre Zagreb, 10000 Zagreb, Croatia; 5 Department of Clinical and Molecular Microbiology, School of Medicine, University of Zagreb, University Hospital Centre Zagreb, 10000 Zagreb, Croatia; abudimir@kbc-zagreb.hr

**Keywords:** dental implant, dental implant–abutment design, implant–abutment connection, microbial colony count, peri-implantitis

## Abstract

Implant–abutment connection (IAC) is a key factor for the long-term success and stability of implant-supported prosthodontic restoration and its surrounding tissues. Misfit between prosthodontic abutment and implant at the IAC leads to technical and biological complications. Two kinds of prosthodontic abutments are currently available on the market: original and third-party abutments. The aim of this pilot study was to test and compare the internal fit (gap) at the implant–abutment interface depending on the abutment fabrication method based on microbial leakage in static conditions and the need for the use of gap sealing material. Two groups of 40 implants were formed on the basis of the type of abutment. In each of the groups of two implant systems, two subgroups of 10 implants were formed. The tested subgroups consisted of 10 implants with sealing material and a negative control subgroups consisting of 10 implants without any sealing material. The test material, GapSeal (Hager and Werken, Duisburg, Germany) was applied in the test subgroups. The implant–abutment assemblies were contaminated with a solution containing *Staphylococcus aureus* and *Candida albicans* for 14 days under aerobic conditions. Results showed that there was no statistically significant difference regarding the microbial leakage between the original and third-party custom-made abutments, regardless of the use of sealing material. It can be concluded that the abutment fabrication method has no significant influence on sealing efficacy regarding the bacterial and fungal leakage in static conditions.

## 1. Introduction

Implant-prosthodontic therapy is an established treatment modality in dental practice that provides high success rates [1]. Implant–abutment connection (IAC) is recognized as a crucial factor for the long-term success and stability of implant-supported prosthodontic restoration and its surrounding tissues, with emphasis on benefits of original abutments [2]. Misfit between such components presents a significant concern because it may lead to mechanical and biological complications [3]. The most common and highly researched biological complication is peri-implantitis, which is influenced by plaque accumulation at the level of the IAC [4]. The presence of a microgap is unavoidable in two-piece implants, and it is precisely this narrow space that makes a small reservoir of microorganisms interfering with the health of the peri-implant tissue [4]. This space is considered to be a critical area in microbial colonization, and also a starting point for peri-implant marginal bone loss [5]. Different implant systems use different designs for the IAC, with the main purpose of microleakage prevention and consequential inflammation of peri-implant tissues. They can be classified as internal or external, with internal being the most commonly used. The internal IAC can be further divided into clearance-fit (or straight), conical, and mixed [2]. However, possible production imprecision and dynamic masticatory load can result in the aforementioned presence of a microgap and micromotion at the IAC, which directly or indirectly might cause technical damage [2]. Even though there is no evidence of complete prevention of miocrobial infiltration through the IAC, there are constant efforts to achieve a tight connection between prosthodontic abutment and implant fixture [6]. The microgap varies between 10 and 135 μm according to different implant systems [6,7]. This is a wide range of values and, moreover, refers to original prosthetic abutments. Two kinds of prosthodontic abutments are currently available on the market for implant restorative procedures: original and third-party abutments [8]. The industry claims that the original parts are better in terms of fit and reduced microleakage [8]. Given the vast possibilities for combinations of variables in implant-prosthodontic rehabilitation, the abutment fabrication method should be carefully evaluated. Regarding these facts, there are materials on the market that are declared to seal the gap at the IAC in order to eliminate microleakage, thus reducing or eliminating biological complications [9]. GapSeal (Hager and Werken, Duisburg, Germany) is such a material, and is based on a highly viscous silicone matrix with thymol. It remains durably viscous and can be removed only by ethanol or by mechanical means. Considering the given information, it should provide long-term protection, avoiding auto- and re-infections by possible microbial accumulation at the IAC [10]. 

Currently, only a limited number of investigations comparing the leakage of original and third-party abutments with the internal type of IAC are available. Therefore, the purpose of this study was to test and compare the internal fit (gap) at the IAC depending on the abutment fabrication method (original and third-party) based on bacterial and fungal leakage in static conditions. A comparison was performed for both straight and conical types of IAC. Additionally, the antimicrobial efficacy and need for the use of gap sealing material was tested. 

The null hypothesis was that the abutment fabrication method would have no influence on the internal fit at the IAC, regardless of the connection type and use of a sealing agent.

## 2. Materials and Methods

### 2.1. Study Design

This microbiological in vitro pilot study was approved by the Ethics Committee of the School of Dental Medicine University of Zagreb (protocol code: 05-PA-30-XII-12/2019 on 5 December 2019) and performed at the laboratory of the Department of Clinical and Molecular Microbiology, University Hospital Centre Zagreb. The microbiological preparation and sampling methodology itself was developed based on recent pilot study by Smojver et al. [9]. The developed protocol has been tested repeatedly, in particular for static in vitro test conditions.

A total of 80 titanium dental implants were used in the study, of which 40 were GC Aadva Standard implants (GCTech.Europe GmbH, Breckerfeld, Germany), with a conical type of connection, and 40 were Zimmer Tapered Screw-Vent implants (Zimmer Biomet Dental, Palm Beach Gardens, FL, USA) with a straight type of connection. The implants were divided into two groups each, regarding the type of prosthetic abutment (A and B). 

Group A consisted of 20 GC Aadva Standard implants (GCTech.Europe GmbH, Breckerfeld, Germany) of 4.0 mm diameter and 20 Zimmer Tapered Screw-Vent implants (Zimmer Biomet Dental, Palm Beach Gardens, FL, USA) of 4.1 mm diameter, both connected to their respective original factory-made prosthodontic abutments.

**Figure 1:**
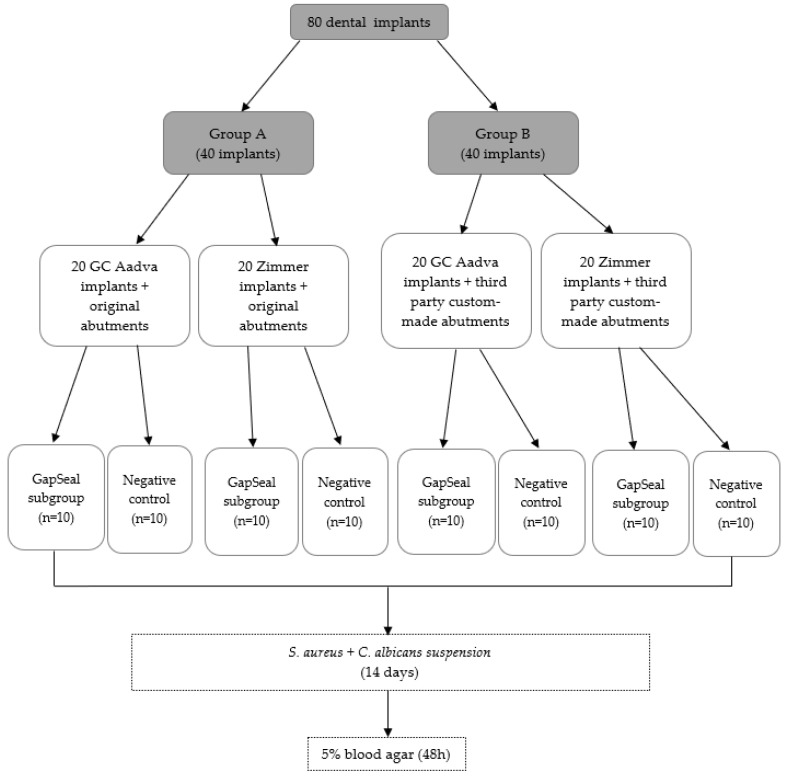
Flow chart illustrating the study design and division of the groups.

Group B consisted of 20 GC Aadva Standard implants of 4.0 mm diameter and 20 Zimmer Tapered Screw-Vent implants of 4.1 mm diameter, both connected to respective third-party custom-made prosthodontic abutments. The abutments were designed in Exocad Galway 3.0 (Exocad GmbH, Darmstadt, Germany). Computer-aided design (CAD) data were sent to computer-aided manufacturing (CAM) software (Mayka Dental 5.1, PicaSoft, Vierzon, France) and then to a Yenadent DC40 milling machine (Yenadent, Vierzon, France). The abutments were milled from a Colado CAD Ti5 (Ivoclar Vivadent AG, Schaan, Liechtenstein) titanium alloy. In each of the groups (A and B), four subgroups of 10 implants were formed. Ten implants per group were required for the study according to the statistical power analysis. The two tested subgroups consisted of 10 Zimmer and 10 GC implants with sealing material and two negative control subgroups consisted of 10 Zimmer and 10 GC implants without any sealing material. GapSeal gel (Hager and Werken, Duisburg, Germany) was used as a sealant. According to the results obtained in the recent study by Smojver et al. [7], it showed the highest values in microbial leakage prevention, so it was the material of choice in this study. (Figure 1)

### 2.2. Preparation of the IAC

**Figure 2:**
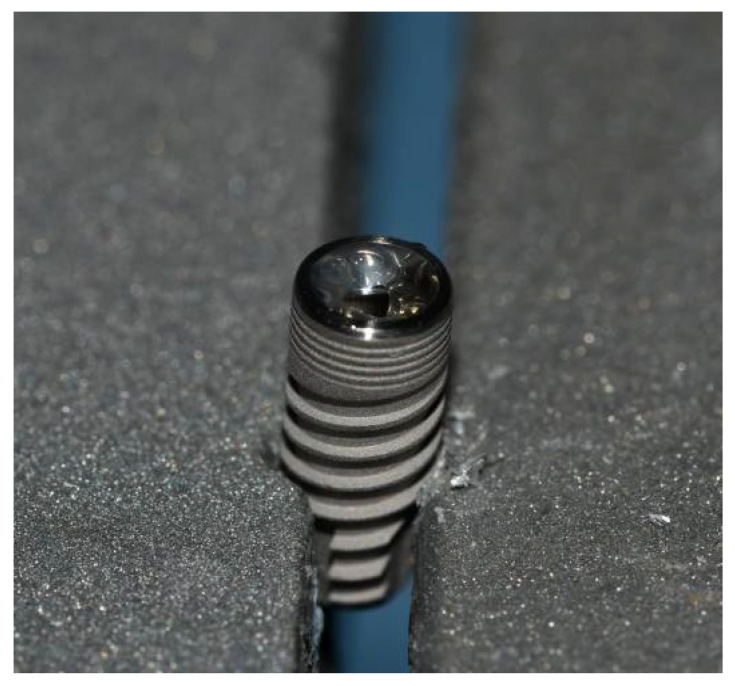
Dental implant fixed in a sterile stainless-steel clamp.

Each dental implant and original complementary abutment were removed from their commercial sterile packaging. Custom-made third-party abutments were sterilized in Euroklav 23 VS+ (Melag, Berlin, Germany) before use. All dental implants were placed in a strictly vertical position in a sterile stainless-steel clamp using sterile stainless-steel forceps (Henry Schein, Melville, NY, USA). Then, they were fixed in the clamp that allowed for a firm swivel action when tightening the prosthetic abutment to the values recommended by the respective manufacturer (20 N/cm for GC Aadva Standard and 30 N/cm for Zimmer Tapered Screw-Vent implants). The clamp also kept the implants in the desired vertical position (Figure 2). 

**Figure 3:**
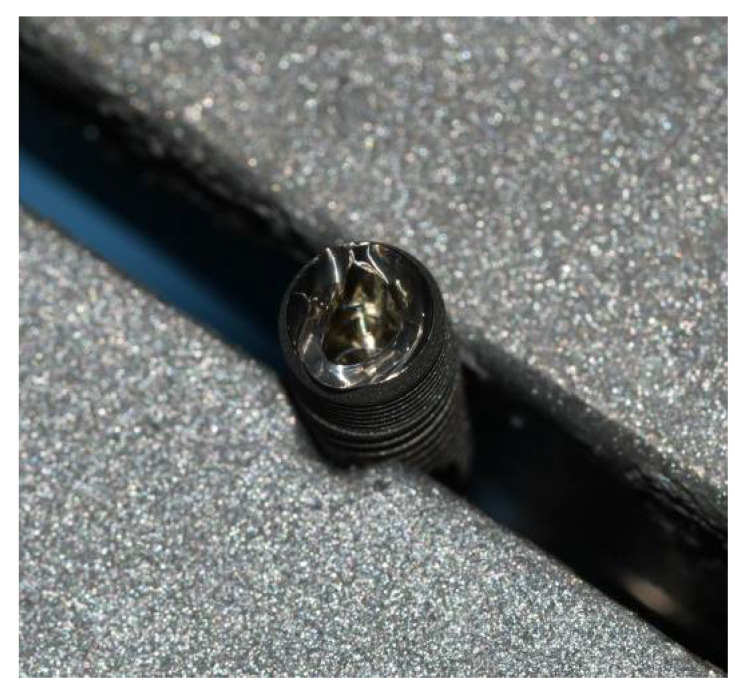
GapSeal gel applied on the implant.

**Figure 4:**
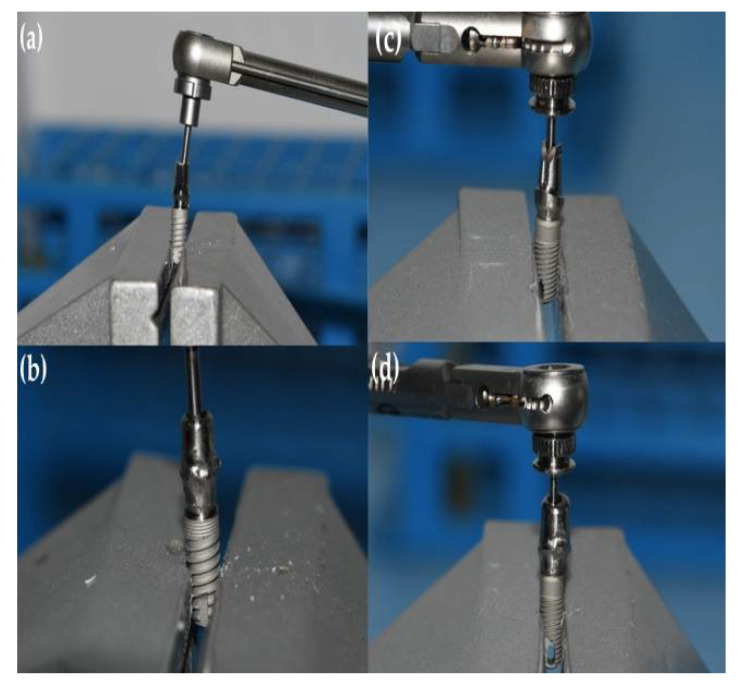
Tightening of the prosthetic abutment. (**a**) GC Aadva Standard implant with original abutment; (**b**) GC Aadva implant with third-party custom-made abutment; (**c**) Zimmer Tapered Screw-Vent implant with original abutment; and (**d**) Zimmer Tapered Screw-Vent implant with third-party custom-made abutment.

Preceding the installation of the prosthetic abutment, a sterile micropipette (Merck KGaA, Darmstadt, Germany) was used to add 0.3 µL of sterile brain heart infusion (BHI) broth (calf brains (12.5 g/L), beef heart infusion solids (5.0 g/L), D-glucose (2.0 g/L), proteose peptone (10.0 g/L), disodium hydrogen phosphate (2.5 g/L) and sodium chloride (5.0 g/L) at a pH 7.4 ± 0.2 and 25 °C to the implants as a non-selective nutrient media in case of bacterial and fungal penetration. GapSeal (Hager and Werken, Duisburg, Germany) was applied to the internal surface of the implants (Figure 3) in the tested subgroups, while the negative control subgroups did not receive the treatment with sealing material. Regardless of sealant use, prosthetic abutments were installed according to the manufacturer’s recommendation (Figure 4).

### 2.3. Contamination of Implant–Abutment Interfaces

**Figure 5:**
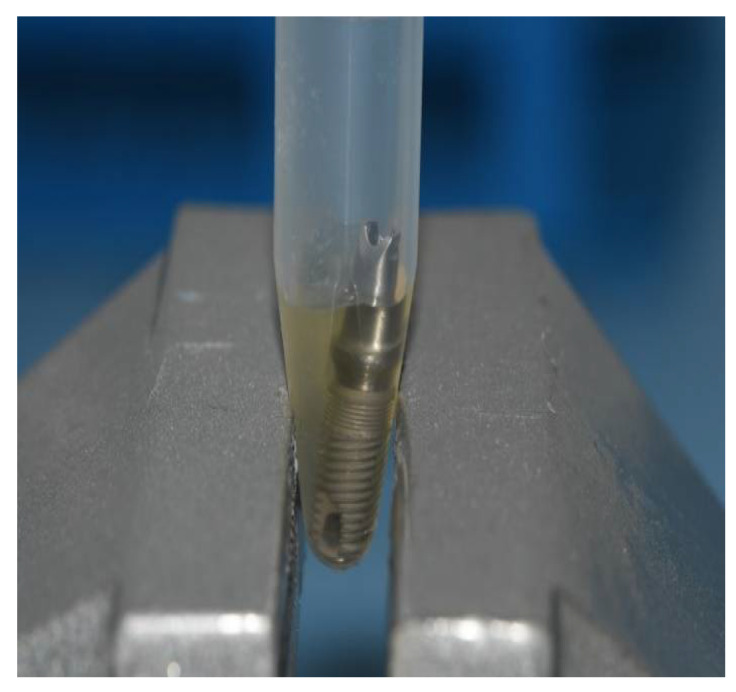
Implant–abutment assembly immersed in bacterial and fungal joint suspension.

Dental implants were contaminated by *Staphylococcus aureus* and *Candida albicans* strains isolated from a clinical sample at Clinical Hospital Centre Zagreb. Firstly, bacterial and fungal strains had been grown separately in Columbia Agar for 72 h following the preparation of separated bacterial and fungal suspensions using thioglycolate broth. They were then mixed together in a joint suspension. An optical densitometer (Densimat, Biomerieux, Marcyl’Etoile, France) was used to set a density of 600 nm, which is equivalent to 1 × 10^8^ colony forming units per milliliter (CFU/mL). All dental implants with installed prosthetic abutments (implant–abutments assemblies) were immersed in 300 µL of mixed bacterial and fungal joint suspension for 14 days under aerobic conditions with an incubation temperature of 35 °C (Figure 5). The suspension contained *S. aureus* and *C. albicans* at a density of 0.5 McFarland.

The abutment screw access hole remained above the level of the suspension to eliminate the impact of the penetration of the contaminated suspension along the fixation screw itself.

**Figure 6:**
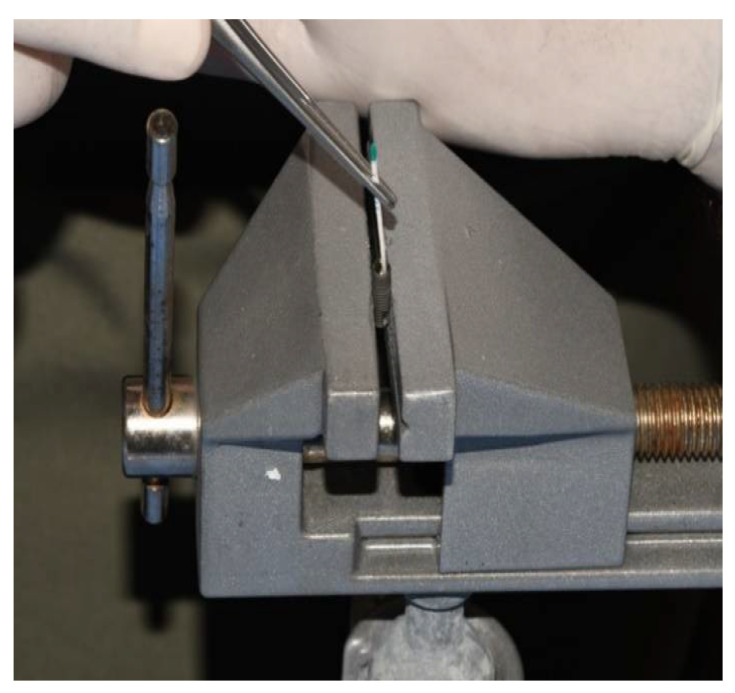
Sampling the implants with paper points.

**Figure 7:**
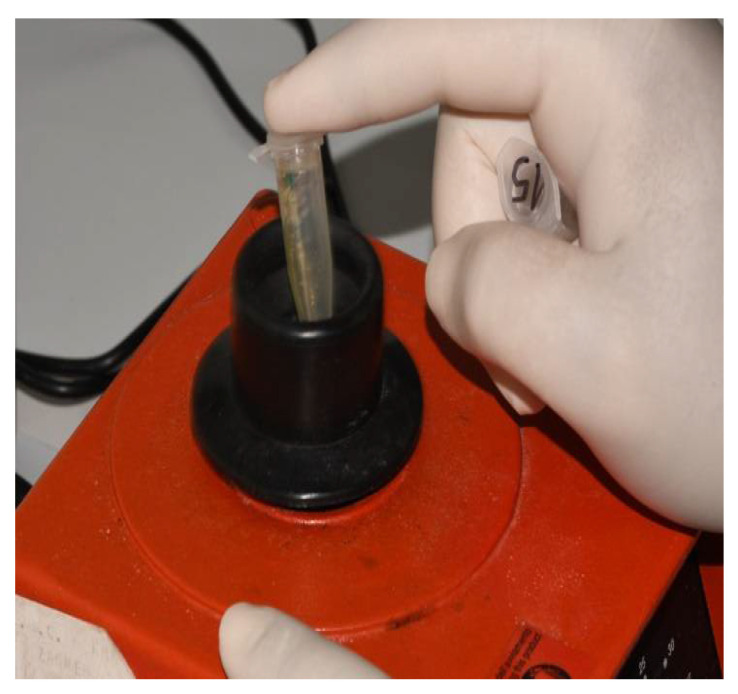
Vortexing.

The implant–abutment assemblies were removed from Eppendorf tubes after 14 days using sterile forceps, following immersion in 70% ethanol for up to 3 min to prevent external contamination. Then, the samples were dried with sterile gauze and put in a sterile clamp. They were carefully disassembled in a strictly vertical position. After the abutments were removed, samples were taken from the internal surfaces of the implants using three sterile paper points (Absorbent points, DENTSPLY Maillefer, Tulsa, OK, USA) (Figure 6), which were then immersed in the Eppendorf tubes containing 0.5 mL of sterile phosphate buffered saline (PBS) solution. The tubes with paper points were inserted into a vortex mixer (Corning^®^ LSE™ vortex mixer, Corning, NY, USA) for 60 s to extract bacterial and fungal cells (Figure 7).

**Figure 8:**
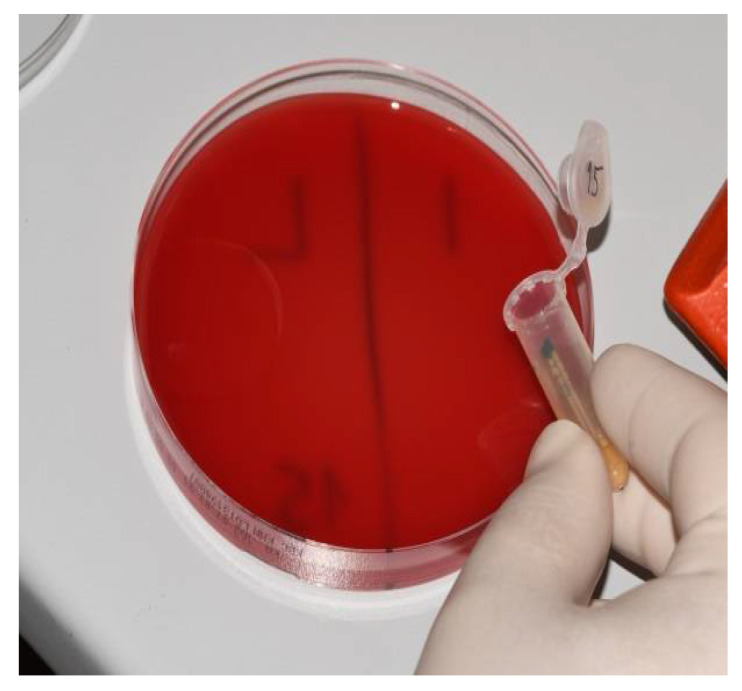
Application of the sample on to 5% blood agar.

**Figure 9:**
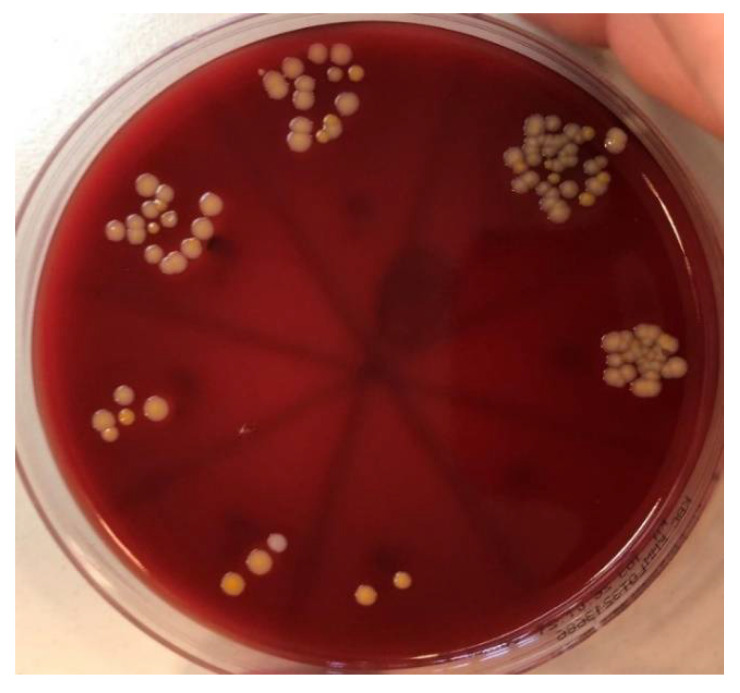
Colonies of *Staphylococcus aureus* and *Candida albicans* on 5% blood agar.

Samples of the tube contents were applied on to 5% blood agar and incubated for 48 h at 37 °C (Figure 8). The resulting colonies were then identified, and quantification was performed. For each sample, the CFU/mL was counted. A MALDI Biotyper (Bruker Daltonics, Hamburg, Germany) was used to verify macroscopically distinctive colonies (Figure 9), and the obtained results underwent further analysis. 

### 2.4. Statistical Analysis

Statistical analysis was performed using Fischer’s exact test, with the traditional level of statistical significance set at *p* < 0.05. Statistical calculation was performed using MedCalc software version 20.014 (Ostend, Belgium).

## 3. Results 

The results were determined based on a frequency of bacterial or fungal microleakage. The presence of *S. aureus* or *C. albicans* signifies a positive result, and complete absence of these bacteria signified a negative result. 

According to the frequencies of bacterial and fungal leakage (Table 1 and Table 2), the third-party custom-made prosthodontic abutments were compared to the original factory-made prosthodontic abutments with regard to infection with Staphylococcus spp. and Candida spp. (Table 3) using the *p*-values of Fisher’s exact test. The abutment fabrication method had no influence on the internal fit at the IAC regarding microleakage since the *p*-values of Fisher’s exact test were greater than the set level of significance (*p* > 0.05), with the lowest *p*-value being 0.4737 (Table 3). Furthermore, there was no statistically significant relationship between the original and third-party abutments with respect to the type of connection, since *p*-values changed by comparable, statistically non-significant amounts in both GC (conical connection) and Zimmer (straight connection) models (Table 3). 

There was no statistically significant relationship between the original and third-party abutments regarding microleakage when gap sealing material was used (Table 4). Data in Table 4 suggest there was more of an impact with sealing material usage in GC implants when compared with Zimmer implants (*p* = 0.0867 for Staphylococcus aureus in GC and *p* = 0.2105 in Zimmer implants), although it was not statistically significant.

**Figure 10:**
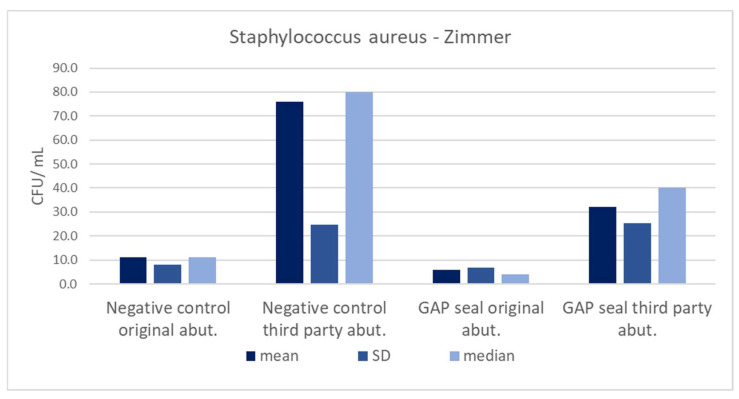
Mean counts of *Staphylococcus aureus* detected on the internal surface of Zimmer implants depending on the abutment fabrication method (original and third-party) and the need for the use of the gap sealing material.

**Figure 11:**
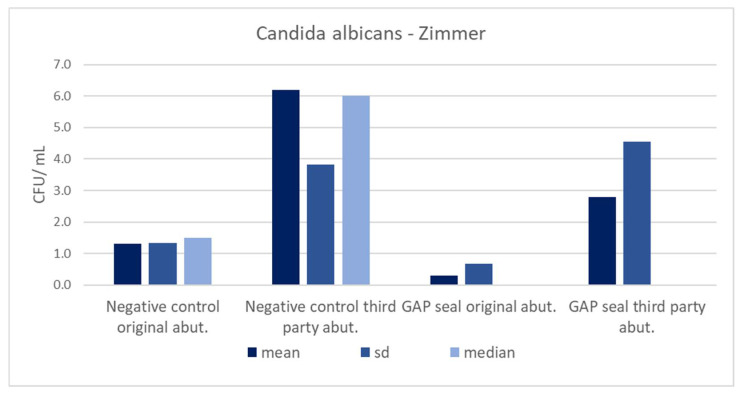
Mean counts of *Candida albicans* detected on the internal surface of Zimmer implants depending on the abutment fabrication method (original and third-party) and the need for the use of the gap sealing material.

**Figure 12:**
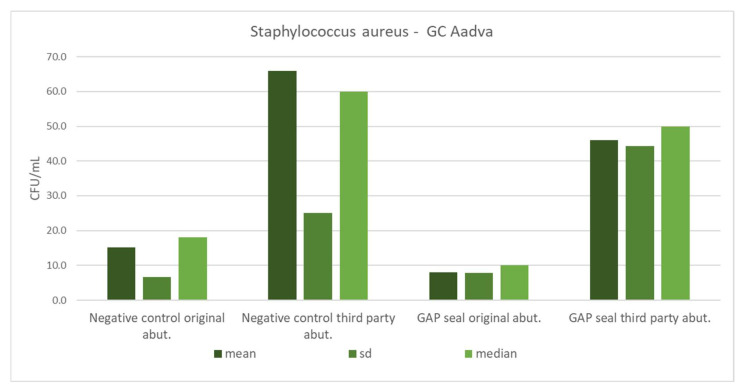
Mean counts of *Staphylococcus aureus* detected on the internal surface of GC implants depending on the abutment fabrication method (original and third-party) and the need for the use of the gap sealing material.

**Figure 13:**
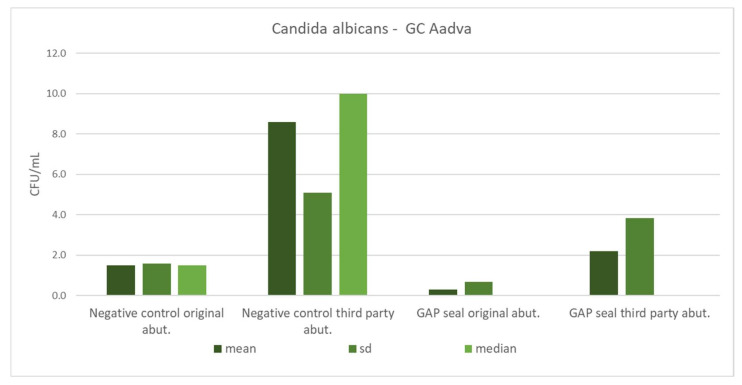
Mean counts of *Candida albicans* detected on the internal surface of GC implants depending on the abutment fabrication method (original and third-party) and the need for the use of the gap sealing material.

Table 5 shows the mean counts of *S. aureus* and *C. albicans* and the influence of different types of connections, abutments, and usage of sealing material on the amount of leaked microbiota. The microbial counts from Table 5 are separately presented in column charts for both GC and Zimmer implants (Figure 10, Figure 11, Figure 12 and Figure 13). There were no significant differences in leaked counts between different types of connections, abutments and with or without sealing material.

## 4. Discussion

The presented in vitro study tested and compared the gaps of the straight and conical IACs depending on the abutment fabrication method based on bacterial and fungal leakage in static conditions, as well as the antimicrobial efficacy of the sealing material. The null hypothesis was accepted, with findings that the prosthodontic abutment fabrication method was not crucial for successful implant-prosthodontic therapy regarding microbial leakage at the IAC in static conditions. Understanding the pathogenesis of peri-implant diseases, the fabrication method of prosthodontic abutments, and the biomechanical role of IAC is of utmost importance in achieving successful clinical results in implant-prosthodontic therapy. 

Considering the finding that bacterial composition of the biofilm formed on dental implants closely resembles that of the neighboring teeth, a switch from peri-implant health to peri-implant mucositis is therefore comparable to gingivitis in terms of bacterial flora [11]. The same postulate is applied in transition to peri-implantitis, which is accompanied by anaerobic species that are commonly found in periodontitis [12]. The biofilm formed around the dental implants is initially dominated by Gram-positive cocci, but eventually shifts to Gram-negative anaerobic and facultatively anaerobic bacteria, such as *Aggregaticabacter actinomycetemcomitans*, *Porphyromonas gingivalis*, *Prevotella intermedia* and *Fusobacterium nucleatum* [13]. Moreover, it was observed that peri-implantitis is often associated with opportunistic pathogens (*Staphylococcus* spp.) and fungal organisms (*Candida* spp.) [14]. Significantly higher counts of *S. aureus* and *S. anaerobius* were detected in implants with peri-implantitis when compared to those of healthy implants [15]. The oral microbiome has more than 100 fungal species, and *C. albicans* plays an important role in the formation and stabilization of biofilm, consequently enabling the development of peri-implant mucositis and peri-implantitis [16]. In addition, *C. albicans* and *S. aureus* are rarely associated with periodontal disease, but possess the ability to attach themselves to titanium surfaces [17]. Taking these findings into consideration, it was decided that dental implants in this study would be contaminated with *S. aureus* and *C. albicans*, as they are the most important microorganisms that cause inflammation of the soft and hard tissues around dental implants. 

Although the differences in microbial leakage between the original and non-original third-party prosthodontic abutments were not statistically significant, non-original third-party abutments showed a more frequent prevalence of infection through the IAC. This result is in accordance with findings from a study by Alonso-Pérez et al. [18]. They concluded that laser-sintered non-original abutment gaps were within the clinically acceptable range of discrepancy. On the other hand, the same authors, in another study, found that original abutments were highly superior to non-original certified abutments in dynamic conditions, but no statistically significant differences were found in static load behavior [19]. It was also observed that the use of non-original abutment components with original Astra Tech implants showed significant leakage at the IAC in static conditions when compared to the use of original prosthetic abutments from same manufacturer [20]. Since the aforementioned study was also performed in static conditions, it is important to highlight that the results were contrary to the results of this study. From a recent systematic review of in vitro studies by Tallarico et al. [8], it was concluded that the original abutments were superior in terms of marginal accuracy, mechanical outcomes and microleakage in the majority of included studies. Nevertheless, they pointed out that in vitro studies had a high risk of bias, and the outcomes reported in these systematic reviews should be carefully interpreted. According to some authors [21,22], abutment screw closing torque can influence the increased microleakage, and the severity of leakage has an inverse correlation with closing torque. Thus, it is of utmost importance to install the prosthetic abutment to the manufacturer’s recommendation. In daily clinical practice, non-original abutments are often selected for financial reasons. Higher leakage values and possible negative mechanical outcomes could be related to many issues that do not allow for exact replication of components, resulting in discrepancies in the dimensions, shape, and design of connecting surfaces. These micromovements at the IAC cause a pumping effect that transports microorganisms from the exterior to the interior surface of the implant and vice versa, creating a vicious circle that results in ongoing infection. In addition to biological issues, further transition of forces from IAC to the implant itself increases the stress on marginal bone level [20]. Precision level and quality control of materials during the manufacturing process are other important factors that must be considered [8].

Further analysis of the results of this study showed that the use of sealing material did not make a statistically significant difference in microleakage at the implant–abutment interface compared to those without sealant. However, GapSeal reduced the amount of leaked microbiota, especially in combination with GC Aadva Standard implants. These improvements were not statistically significant, but gave valuable insights for further studies. A complete hermetic seal at the IAC is not achievable, according to the contemporary literature [9,23,24]. The difference between original and third-party abutments regarding microleakage when sealing material is used is inevitably related to internal fit at the IAC. Therefore, it is precisely the marginal accuracy and appropriate design of non-original abutments that play vital roles in the elimination of microleakage. Smojver et al. [9] and Biscoping et al. [25] confirmed that the presence of the sealing agent may be useful in reducing microbial infiltration into the implants. It was concluded that the application of sealing material before abutment connection may reduce the bacterial and fungal populations of the peri-implant, but a complete seal against bacterial infection was not formed at the implant–abutment interface when using different sealing materials (GapSeal, Oxysafe and Flow.sil) [9]. Biscoping et al. [25] found that the tested sealing materials (Clorhexamed 1% gel and Berutemp) did not influence the gap at the IAC, but the same materials also decreased the torque necessary for loosening the abutment screws. This finding suggests that sealing agents might contribute to negative mechanical outcomes affecting the reverse torque values. Seloto et al. [26] observed that sealing gel (Loctite 2400) promoted lower vertical misfit values at the IAC and preload maintenance of screw-retained prostheses after mechanical cycling. Furthermore, Yu et al. [27] concluded that the GapSeal material reduced microleakage at the IAC after dynamic loading and reported evident abutment screw thread wear protection in three different implant systems with internal conical connection. It is important to emphasize that dynamic conditions in which that study was conducted contributed to different outcomes and plausible major advantages of sealing material usage when compared to those in static conditions. 

Additionally, the presented results did not show a statistically significant difference between original and third-party abutments regarding the type of connection. There is a lack of studies that compare these two types of abutments and the influence of connection type on microleakage at the same time. Considering the type of connection alone, there are various studies observing the connection type with minimal microleakage. De Sousa et al. [28] observed that the external hexagonal connection was more effective than the Morse Taper connection against microbial infiltration for dual species biofilms. Conversely, Quirynen et al. [29] described that connections with an external six-fold design were more prone to microbial invasion. There is also evidence that implants with an internal hexagonal connection are more resistant to bacterial leakage under dynamic loading [30]. The superiority of a conical connection regarding seal performance, gap formation and mechanical stability has also been demonstrated in the literature due to the homogeneous spread of the load [31]. Therefore, the aforementioned studies support the results of this study and, although there was no statistically significant difference between a conical connection and straight connection, GC Aadva implants with a conical connection had slightly better results in combination with sealing material regarding microleakage. 

Within the limitations of this in vitro study, primarily a static testing condition and sample size, interesting scientific results were found. However, a larger sample size is needed in future studies, considering the high standard deviation values in the results, and further extensive clinical research should be conducted to assess the outcomes of this study.

## 5. Conclusions 

According to the presented results, the abutment fabrication method had no significant influence on the sealing efficacy of the IAC regarding the leakage of bacteria and fungi. Considering the discussed limitations of this study, third-party custom-made abutments represent a viable solution from a microbiological point of view. It is not mandatory to use sealing material, since there was no statistically significant difference in microleakage relative to the presence of the sealing material regardless of the type of abutments. These findings gave important evidence to support studies that would provide more clinical evidence about the long-term outcomes of custom-made abutments and their sealing efficacy. 

## Figures and Tables

**Table 1:** The frequencies of bacterial and fungal microleakage (Zimmer Tapered Screw-Vent implants).

Microbe	Original Abutments [%]	Third-Party Custom-Made Abutments [%]	Original Abutments with GapSeal [%]	Third-Party Custom-Made Abutments with GapSeal [%]
*Staphylococcus aureus*	80.00 (8/10)	100.00 (10/10)	50.00 (5/10)	70.00 (7/10)
*Candida albicans*	60.00 (6/10)	80.00 (8/10)	20.00 (2/10)	30.00 (3/10)

**Table 2:** The frequencies of bacterial and fungal microleakage (GC Aadva Standard implants).

Microbe	Original Abutments [%]	Third-Party Custom-Made Abutments [%]	Original Abutments with GapSeal [%]	Third-Party Custom-Made Abutments with GapSeal [%]
*Staphylococcus aureus*	90.00 (9/10)	100.00 (10/10)	60.00 (6/10)	60.00 (6/10)
*Candida albicans*	60.00 (6/10)	80.00 (8/10)	20.00 (2/10)	30.00 (3/10)

**Table 3:** Comparison of Fisher’s exact test values for microleakage between original and third-party custom-made prosthodontic abutments.

Implant	Zimmer		GC	
Fisher Exact Test (*p*-Values)	*Staphylococcus aureus*	*Candida albicans*	*Staphylococcus aureus*	*Candida albicans*
Original prosthodontic abutment	H0 accepted (0.4737)	H0 accepted (0.6285)	H0 accepted (0.5000)	H0 accepted (1.0000)
Third-party custom-made prosthodontic abutment	H0 accepted (0.6499)	H0 accepted (1.0000)	H0 accepted (0.6285)	H0 accepted (1.0000)

* Statistically significant (*p* < 0.05). ** Null hypothesis: the abutment fabrication method would have no influence on the internal fit at the IAC, regardless of the connection type and use of a sealing agent.

**Table 4:** Comparison of Fisher’s exact test values for microleakage with and without application of sealing material (GapSeal).

Implant	Zimmer		GC	
Fisher Exact Test (*p*-Values)	*Staphylococcus aureus*	*Candida albicans*	*Staphylococcus aureus*	*Candida albicans*
Without sealing material	0.3498	0.1698	0.3034	0.1698
With sealing material	0.2105	0.0698	0.0867	0.0698

* Statistically significant (*p* < 0.05).

**Table 5:** Mean counts of *Staphylococcus aureus* and *Candida albicans* detected on the internal surface of the implants depending on the abutment fabrication method (original and third-party) and the need for the use of the gap sealing material.

	*Staphylococcus aureus*	*Candida albicans*
**Zimmer**	**CFU/mL mean +/− SD (median)**	**CFU/mL mean +/− SD (median)**
Negative control original abut.	11.2 +/− 7.9 (11)	1.3 +/− 1.34 (1,5)
Negative control third-party abut.	76 +/− 24.59 (80)	6.2 +/− 3.82 (6)
GapSeal original abut.	5.8 +/− 6.89 (4)	0.3 +/− 0.67 (0)
GapSeal third-party abut.	32 +/− 25.3 (40)	2.8 +/− 4.54 (0)
**GC Aadva**	**CFU/mL mean +/− SD (median)**	**CFU/mL mean +/− SD (median)**
Negative control original abut.	15.2 +/− 6.68 (18)	1.5 +/− 1.58 (1.5)
Negative control third-party abut.	66 +/− 25.03 (60)	8.6 +/− 5.08 (10)
GapSeal original abut.	8 +/− 7.89 (10)	0.3 +/− 0.67 (0)
GapSeal third-party abut.	46 +/− 44.27 (50)	2.2 +/− 3.82 (0)

## Data Availability

The data presented in this study are available on request from the corresponding author.
